# Giant Cell Arteritis With Atypical Features: Acute Vision Loss and Marked Eosinophilia Mimicking Systemic Vasculitis

**DOI:** 10.7759/cureus.86195

**Published:** 2025-06-17

**Authors:** David Phrathep, Sabrina A Billings, Hamaad Khan, Palak Patel, Michael Mohseni

**Affiliations:** 1 Physical Medicine and Rehabilitation, Mayo Clinic, Jacksonville, USA; 2 Internal Medicine, Mayo Clinic, Jacksonville, USA; 3 Family Medicine, Mayo Clinic, Jacksonville, USA; 4 Emergency Medicine, Mayo Clinic, Jacksonville, USA

**Keywords:** emergency medicine, giant cell arteritis, seronegative eosinophilic granulomatosis with polyangiitis, temporal arteritis, ultrasonography, vasculitis, vision loss

## Abstract

Giant cell arteritis (GCA), also known as temporal arteritis, is a chronic inflammatory vasculitis that affects the large- and medium-sized arteries, which commonly includes the cranial branches of the carotid arteries. Manifestations range from constitutional symptoms, headache, and jaw claudication to transient or permanent monocular vision loss. Because its prognosis is poor if untreated, patients require prompt evaluation and appropriate treatment. We present an atypical case of binocular vision loss in a patient diagnosed with GCA with eosinophilic granulomatosis with polyangiitis. The patient declined a temporal artery biopsy and opted for ultrasonography, which helped clinch the ultimate diagnosis. His symptoms partially improved with high-dose steroids and tocilizumab in the hospital. Our case highlights the diagnostic challenges, appropriate specialist consultations required, and prompt management of GCA with overlapping vasculitis symptoms.

## Introduction

Giant cell arteritis (GCA), also known as temporal arteritis, is a systemic vasculitis affecting mainly large- and medium-sized vessels, typically occurring in adults older than 50, especially women [1]. It is thought to be a result of innate and adaptive immune responses that cause vessel wall damage and granuloma formation, often seen with pathognomonic multinucleated giant cells [2]. This inflammatory response leads to vessel stenosis and ischemia, presenting as headaches, scalp tenderness, jaw claudication, and, most critically, vision loss. GCA patients have a one-year mortality of 6.4% and have been shown to be at greater risk of mortality from concomitant disease [3].

GCA should be considered when patients present with the common cranial symptoms of temporal headache, jaw claudication, and vision changes. Extracranial symptoms like pulselessness and limb claudication may also indicate large vessel involvement and should be investigated promptly. Polymyalgia rheumatica is strongly associated with GCA [2]; therefore, symptoms of stiffness in the neck, shoulders, or pelvic girdle, alongside myalgias and other constitutional symptoms, should be thoroughly investigated for concurrent GCA symptoms.

Diagnosis is primarily clinical, and laboratory findings include elevation of inflammatory markers such as C-reactive protein (CRP) and erythrocyte sedimentation rate (ESR) alongside hematologic changes like neutrophilia, thrombocytosis, and normocytic anemia from chronic inflammation [4]. Although temporal artery biopsy remains the gold standard for diagnosis, additional non-invasive modalities, including magnetic resonance imaging (MRI) and ultrasound, have been utilized more frequently in recent years [5]. Failing to investigate or intervene on suspected GCA early in the course of illness can result in permanent ischemic damage and irreversible vision loss, as well as stroke or aortic aneurysm [6].

The treatment of suspected GCA is immediate administration of high-dose corticosteroids to prevent permanent changes like vision loss [4]. The use of adjunctive immunotherapies such as tocilizumab or methotrexate has demonstrated success, especially in patients with glucocorticoid-induced adverse reactions [5]. These steroid-sparing agents should be considered in patients who may not tolerate high-dose corticosteroid treatment or patients for whom corticosteroid treatment is not completely effective in controlling GCA symptoms [5]. Our case describes an atypical presentation of GCA in the setting of eosinophilic granulomatosis with polyangiitis, demonstrating the importance of recognizing GCA symptoms and initiating therapeutic measures immediately.

## Case presentation

A 60-year-old male presented to the emergency department (ED) with acute-onset bilateral vision loss. The symptoms began as blurred vision the evening prior to presentation and worsened overnight, resulting in complete vision loss in the left eye and increased vision loss in the right eye by the time he reached the ED the next morning. The patient reported taking a new cannabidiol supplement the previous night but denied any other new medications, headaches, history of clotting disorders, or previous stroke. The patient’s past medical history included hypercholesterolemia, asthma, and squamous cell carcinoma of the scalp treated with Mohs surgery and 5-fluorouracil. He did report a family history of deep vein thrombosis and pulmonary embolism. Additionally, on review of systems, he mentioned recent facial swelling, mild sore throat, subjective fever, rash to the lower extremities, and decreased appetite.

Upon examination, his vital signs were stable as follows: temperature 36.7°C, pulse rate 101 beats per minute, respiratory rate 15 breaths per minute, blood pressure 119/80 mmHg, and pulse oximetry 96% on room air. Physical examination revealed that the patient was in no acute distress. His head was atraumatic, the nose was clear, and the oropharynx was moist. Eye examination revealed normal extraocular movements, but was significant for bilateral pupillary dilation with minimal reactivity to light. Visual acuity testing indicated that the patient could only see dark gray motion in the left eye and was able to discern gross objects and count fingers with the right eye. Skin examination revealed palpable purpuric skin lesions on the lower extremities.

**Figure 1:**
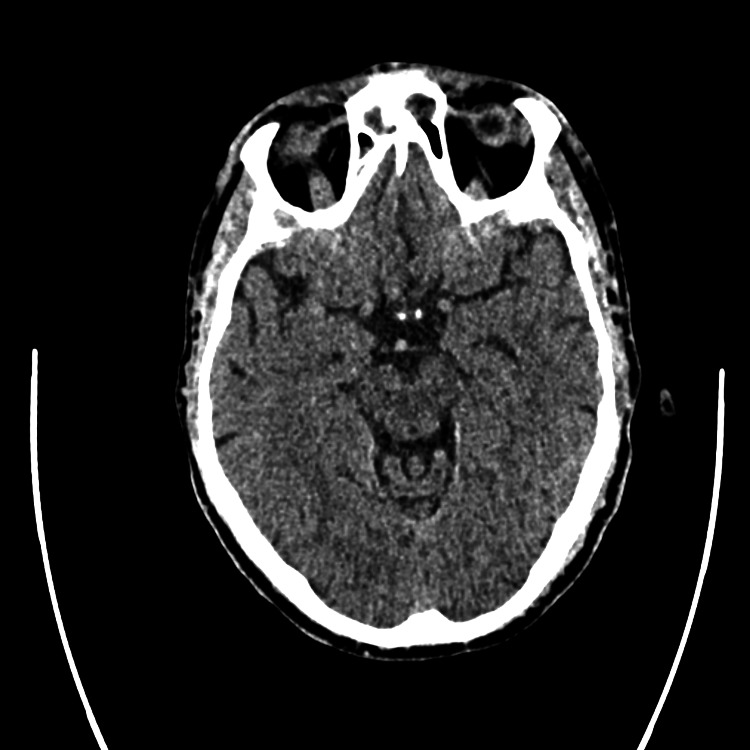
Normal computed tomography of the head without evidence of intracranial hemorrhage.

**Figure 2:**
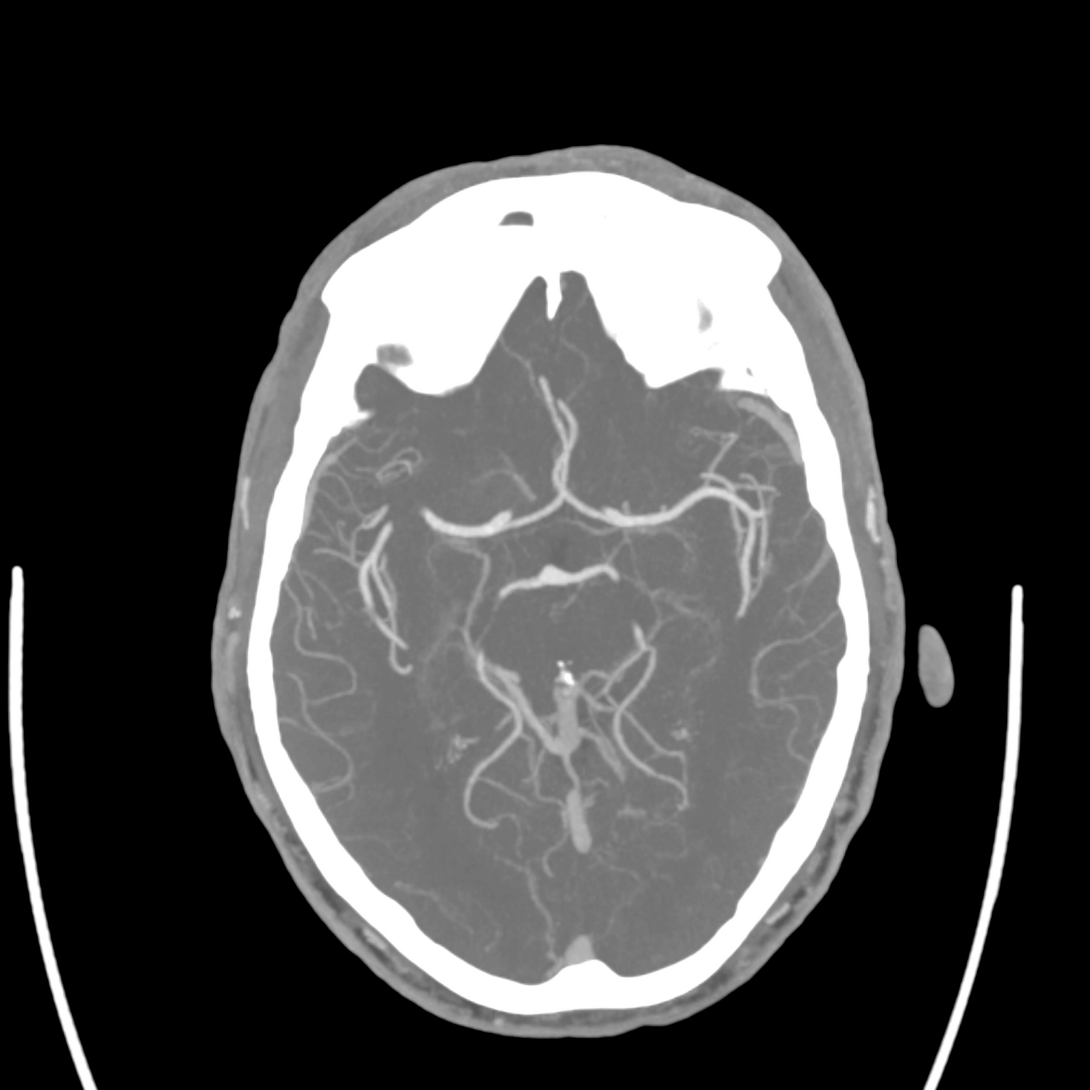
Normal computed tomography angiography without evidence of vessel occlusion or stenosis.

Neurology was consulted due to the acute onset of symptoms and abnormal pupillary findings. The patient underwent an acute stroke protocol workup in the ED. Neurological assessment showed partial hemianopia with a National Institutes of Health Stroke Scale score of 1 for this visual disturbance. Computed tomography (CT) of the head was negative for intracranial hemorrhage (Figure 1), and CT angiography ofthe head and neck was negative for any occlusion or stenosis (Figure 2).

The neurology team recommended admission for further infectious and inflammatory workup. The Hospital Internal Medicine team was involved in the escalation of care, further evaluation, and admission to the hospital.

Laboratory testing was notable for an elevated ESR, elevated CRP, significant eosinophilia, and elevated rheumatoid factor (Table 1).

**Table 1:** Abnormal laboratory testing found during the patient's workup. ESR: erythrocyte sedimentation rate, CRP:  C-reactive protein, CBC: complete blood count Rheumatoid factor was measured on day 2, while all other parameters were measured on day 0.

Laboratory test	Value	Reference range
ESR	128 mm/hr	0-22 mm/h
CRP	185.9 mg/L	<5.0 mg/L
Eosinophilia on CBC	20.20%	1.0-3.0%
Rheumatoid factor	61 IU/mL	<15 IU/mL

Multiple specialists were subsequently involved in this patient's care. Ophthalmology was consulted and assessed the patient for his severely decreased visual acuity. On their evaluation, anterior optic neuropathy was identified. This suggested a differential diagnosis including GCA versus other autoimmune disorders. Given this finding, our rheumatology team was consulted for recommendations on further inflammatory workup and potential use of biologics, such as tocilizumab. Upon the recommendation of the rheumatologists, the treatment plan included starting intravenous methylprednisolone 1000 mg daily, additional laboratory testing including infectious and autoimmune panels, a temporal artery biopsy, an MRI of the orbits, and a lumbar puncture. 

**Figure 3:**
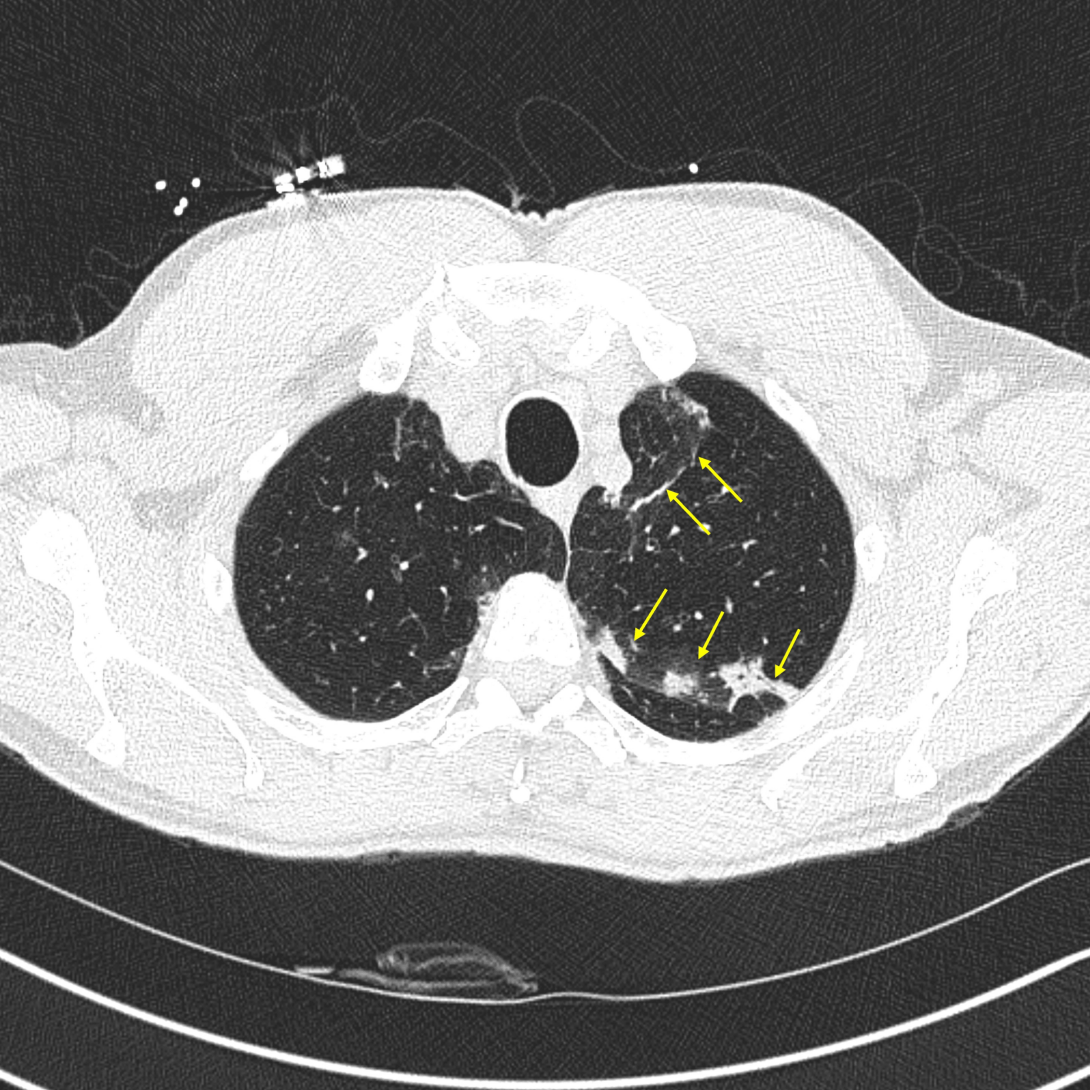
Computed tomography of the chest revealing ground-glass opacities (arrows).

Further imaging in the form of CT of the chest revealed ground-glass opacities and multiple, small (<4 mm) pulmonary nodules (Figure 3). 

**Figure 4:**
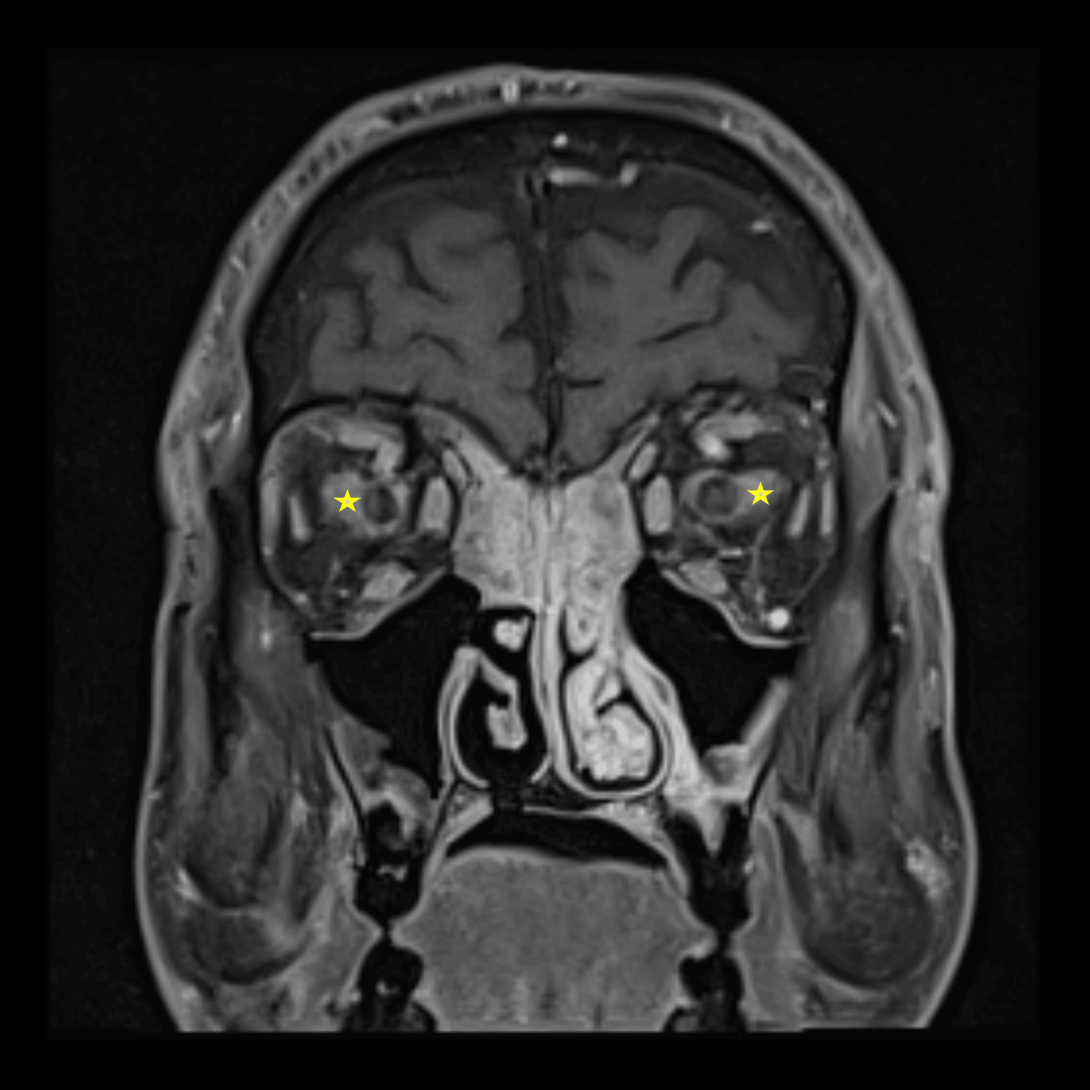
Magnetic resonance imaging of the orbits with stranding of the soft tissue fat near optic nerve insertions (stars).

On MRI of the orbits, soft tissue stranding was noted around the optic nerves bilaterally, along with diffuse paranasal sinus mucosal thickening (Figure 4). 

**Figure 5:**
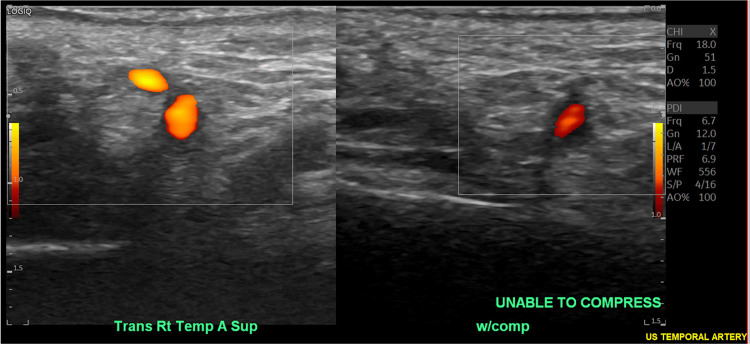
Temporal artery ultrasound revealing lack of compressibility of the right temporal artery (compression in right frame with notable continued arterial flow).

**Figure 6:**
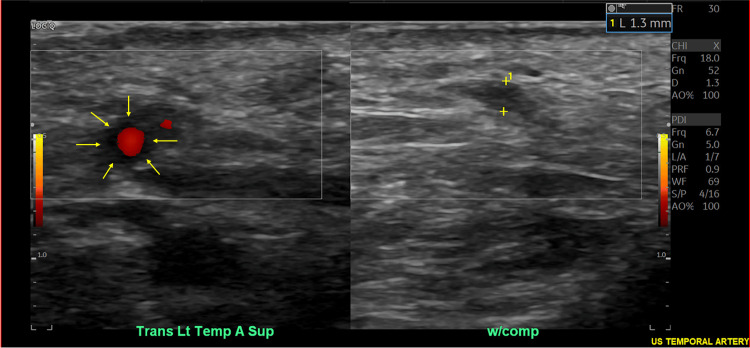
Temporal artery ultrasound revealing "halo sign" of the left temporal artery (left frame, arrows).

These imaging findings supported an underlying inflammatory process. Despite recommendations for a temporal artery biopsy, the patient refused and chose non-invasive diagnostics. A temporal artery ultrasound confirmed lack of compressibility of the right temporal artery (Figure 5) and a distinct "halo sign" surrounding the main left temporal artery (Figure 6), both consistent with GCA.

The patient did acquiesce, however, to a skin biopsy (performed by our dermatology team) of the lower extremity purpuric lesions later during his hospital course. This revealed medium-vessel vasculitis with neutrophilic and eosinophilic inflammation along with early thrombosis. Further evaluations, including magnetic resonance angiography of the chest, abdomen, and pelvis, were unrevealing for extracranial vasculitis. Laboratory and cerebrospinal fluid analysis, including tests for syphilis, cryoglobulins, and serum protein electrophoresis, were all also negative. 

Rheumatology recommended continuing high-dose intravenous steroids followed by a prolonged prednisone taper as follows: three days of intravenous methylprednisolone 1000 mg daily, then switching to oral prednisone at 60 mg with a subsequent prolonged 25-week taper. Moreover, while inpatient, tocilizumab 4 mg/kg was given intravenously for one dose, with plans for outpatient follow-up to determine if the patient would continue with this regimen. The therapies in the hospital resulted in partial improvement in the vision of the right eye, but without significant vision recovery in the left eye. The patient was discharged in stable condition after a four-day hospitalization.

In the outpatient setting, the rheumatologist team recommended starting cyclophosphamide therapy rather than continuing tocilizumab, given that the patient’s presentation was more consistent with seronegative eosinophilic granulomatosis with polyangiitis (EGPA), presenting atypically with temporal arteritis and mimicking polyarteritis nodosa. Trimethoprim-sulfamethoxazole was prescribed for Pneumocystis jirovecii pneumonia prophylaxis in the setting of prolonged high-dose corticosteroid therapy. Additional medications recommended included a proton-pump inhibitor for gastrointestinal protection, along with calcium and vitamin D supplementation to prevent steroid-induced bone loss. The patient continued with regular monitoring in the outpatient setting but unfortunately did not have any additional improvement in vision at a six-month follow-up.

## Discussion

GCA is a rare disease that can have severe complications. The clinical presentation can be typical or atypical (as was the case in our patient). Understanding various presentations of GCA is crucial for improving the clinical complications of this autoimmune disease.

Our patient presented with the distressing cranial manifestation of sudden vision loss bilaterally. GCA can present with extra-cranial manifestations as well, but it can include fever, fatigue, aortic aneurysm, limb claudication, or absent pulses [7]. Vision loss can happen early in the course of the disease and has been found to occur in up to 20% of patients [8]. Vision loss occurs secondary to ischemia, which occurs because of arterial inflammation. The resolution of vision loss can be quite complicated if treatment is delayed. Kokloni et al., in their prospective case-based review of vision loss in GCA, reported that 16% of 339 patients suffered permanent vision loss [8]. When those patients were stratified further, they concluded that patients with visual symptoms prior to steroid initiation were at risk for their vision worsening. Furthermore, another prospective study conducted by Liozon et al. found that no permanent vision loss occurred with either high-dose oral prednisone or pulse-dosed methylprednisolone if there was no established vision loss before treatment initiation [9]. These studies reiterate the importance of treating GCA patients promptly, especially when there is a concern for visual symptoms.

Our patient’s case was more atypical due to the involvement of EGPA. EGPA is a vasculitis syndrome that typically involves small- to medium-sized vessels and is associated with asthma, sinusitis, anti-neutrophil cytoplasmic antibody positivity, and eosinophilia [7]. On the other hand, GCA typically involves medium to large vessels such as the temporal arteries and rarely involves eosinophilia. There are very few case reports describing the association between EGPA and GCA. Nishimura et al. described a patient's case of EGPA that presented with GCA only, which was evidenced by the presence of eosinophils on temporal artery biopsy [10]. The patient in our case report was unique due to the presence of small vessel vasculitis, evidenced by neutrophilic and eosinophilic inflammation on skin biopsy in addition to his temporal arteritis. EGPA diagnosis is more likely when there are more than two organs showing eosinophilic vasculitis involvement [10].

Although our patient did not have a temporal artery biopsy completed, he did have a temporal artery ultrasound with findings consistent with GCA. Diagnosis via ultrasound can be useful in centers that are experienced with this technology [5]. Temporal artery ultrasonography can reveal characteristic findings in individuals with GCA, such as the "halo sign," which is described as hypoechoic, circumferential wall thickening caused by edema (as noted on our patient's temporal artery ultrasound) [11]. Using clinical diagnosis as a standard, the sensitivity and specificity of temporal artery ultrasonography have been reported as high as 67% and 95%, respectively, in one recent meta-analysis [12]. Other than the aforementioned hypoechoic "halo sign," thickening of the vessel wall consistent with mural inflammation may cause a lack of compressibility on temporal artery ultrasonography in GCA patients [13]. Although ultrasound is a non-invasive means of potential diagnosis, the interpretation remains somewhat subjective and dependent on the skill level and experience of the ultrasound technologist. Other diagnostic tools have evolved, such as positron emission tomography and CT imaging, to detect vascular inflammation [5]. 

In summary, vision loss is a cranial manifestation of GCA that should be intervened upon expeditiously with corticosteroids, steroid-sparing agents, or both. When not promptly diagnosed and treated, GCA can cause significant ocular manifestations and complications, most commonly anterior ischemic optic neuropathy [14]. Like many other autoimmune diseases, GCA treatment aims to minimize inflammation and decrease symptom burden. Patients require close surveillance for recurrence of symptoms, and involving a rheumatologist can be pivotal in a patient’s illness trajectory. This case emphasizes the importance of understanding atypical presentations of GCA so that clinicians can be alert to the possible diagnosis early in the course of illness.

## Conclusions

Our case presentation examines an atypical presentation of GCA in the context of EGPA, while also emphasizing the diagnostic challenges that arise when presented with overlapping symptoms of vasculitis syndromes. Despite the atypical nature of this case, recognizing GCA symptoms early allowed the care team to intervene in a timely fashion, preventing permanent vision loss in both eyes. The patient’s decision to decline a temporal artery biopsy spotlights the importance of a flexible, comprehensive approach to diagnosis through non-invasive imaging and laboratory testing. Furthermore, this case emphasizes the need for clinicians to consider GCA in the differential diagnosis even in presentations complicated by eosinophilia and atypical symptoms, as a delayed diagnosis can exacerbate ocular morbidity.
